# Petersen’s space hernia as an immediate complication in a patient undergoing gastric bypass: a case report

**DOI:** 10.1093/jscr/rjae589

**Published:** 2024-09-26

**Authors:** Alberto Michel Macareno, Johanna Betzabe Cobos Román, Rafael Michel Esparza, Jesús Antonio Gil Gamez, Ariana Medina Estrada, Isaac Esparza Estrada

**Affiliations:** Department of Bariatric Surgery, Obesity Not 4 Me, Río Médica, Leona Vicario 1451, 3er. Piso, Zona Urbana Rio Tijuana, 22010 Tijuana, B.C., México; Department of Bariatric Surgery, Obesity Not 4 Me, Río Médica, Leona Vicario 1451, 3er. Piso, Zona Urbana Rio Tijuana, 22010 Tijuana, B.C., México; Department of Bariatric Surgery, Obesity Not 4 Me, Río Médica, Leona Vicario 1451, 3er. Piso, Zona Urbana Rio Tijuana, 22010 Tijuana, B.C., México; Department of Bariatric Surgery, Obesity Not 4 Me, Río Médica, Leona Vicario 1451, 3er. Piso, Zona Urbana Rio Tijuana, 22010 Tijuana, B.C., México; Department of Bariatric Surgery, Obesity Not 4 Me, Río Médica, Leona Vicario 1451, 3er. Piso, Zona Urbana Rio Tijuana, 22010 Tijuana, B.C., México; Department of Bariatric Surgery, Obesity Not 4 Me, Río Médica, Leona Vicario 1451, 3er. Piso, Zona Urbana Rio Tijuana, 22010 Tijuana, B.C., México

**Keywords:** Petersen’s space hernia, roux-en-Y gastric bypass, internal hernia, laparoscopic surgery, preventive techniques

## Abstract

Petersen’s space hernias are common internal hernias following laparoscopic gastric bypass surgery, occurring when intestinal loops protrude through the space between the mesentery of the alimentary limb and the transverse mesocolon. A 43-year-old female with a history of hypertension underwent a revisional Roux-en-Y gastric bypass due to weight regain and severe gastroesophageal reflux disease. Postoperatively, she developed abdominal pain, and an urgent diagnostic laparoscopy was performed, revealing a nearly complete herniation of the alimentary limb through Petersen’s mesenteric defect. Based on these findings, an urgent laparotomy was subsequently performed to reduce the herniated bowel and close the defect. Her postoperative course was uneventful, and she was discharged without complications. Despite the closure of Petersen’s mesenteric defect, vigilance for hernia remains crucial due to the risk of severe complications. Advances in preventive techniques show promise, but prompt diagnosis and intervention are essential for improving patient outcomes.

## Introduction

Petersen’s space hernias are the most common location for internal hernias following laparoscopic gastric bypass surgery. Petersen’s hernia occurs when intestinal loops protrude through the space created between the mesentery of the alimentary limb and the transverse mesocolon. Their diagnosis or even the suspicion thereof warrants urgent surgery. The clinical presentation is seldom specific; it may exhibit symptoms such as acute abdomen with or without vomiting and symptoms of small bowel obstruction. Therefore, a high index of suspicion is warranted for differential diagnosis in patients following Roux-en-Y gastric bypass surgery [1].

We present the case of a patient who required an urgent diagnostic laparoscopy and a subsequent laparotomy 12 hours after hospital discharge, following an initial revisional Roux-en-Y gastric bypass surgery. The herniated intestinal loops were reduced and Petersen’s space was closed despite having been closed during the initial procedure [2].

## Case presentation

A 43-year-old female patient with a history of hypertension was previously treated with a sleeve gastrectomy 9 years ago, starting with an initial BMI of 48.1 and losing 55 kg over the next 4 years. She presented with a 38 kg weight regain and severegastroesophageal reflux disease, prompting revisional surgery. A Roux-en-Y gastric bypass was performed with intraoperative endoscopy confirming patency and adequate hemostasis of the anastomosis.

After an uneventful postoperative course, she was discharged after 48 hours. Eight hours post-discharge, she presented with abdominal pain that began in the mesogastrium and progressively intensified.

On examination, she exhibited antalgic posture, tachycardia at 140 bpm, and a tender mesogastrium without distension or signs of peritoneal irritation.

**Figure 1:**
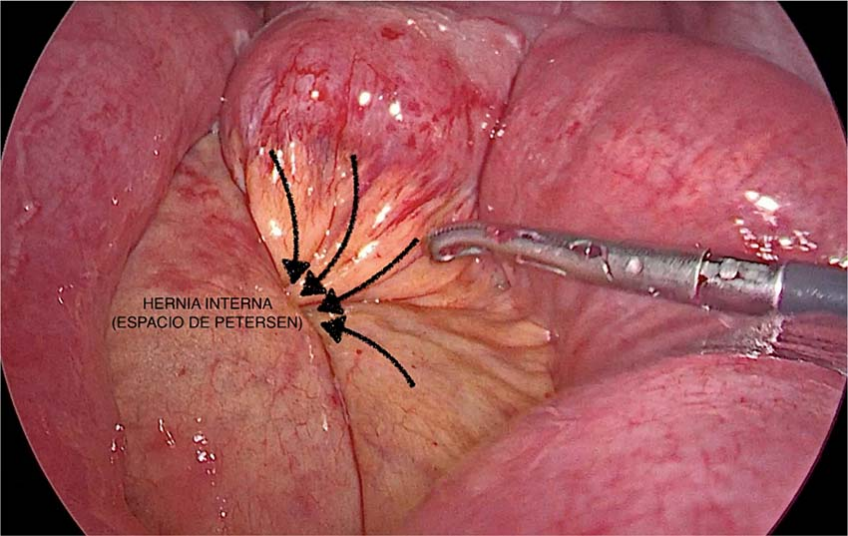
Laparoscopic visualization of Petersen’s hernia.

**Figure 2:**
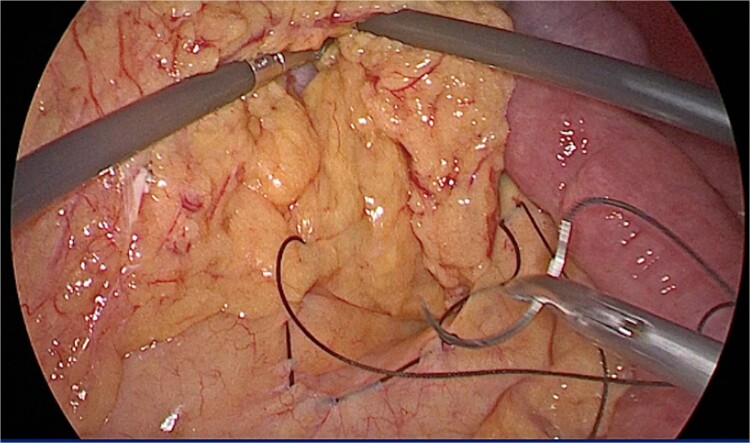
Repair of Petersen’s hernia with 2–0 silk sutures.

An urgent diagnostic laparoscopy revealed dilated bowel loops and nearly complete herniation of the alimentary limb through Petersen’s mesenteric defect (Fig. 1). Due to tissue friability and bowel distension, the procedure was converted to a laparotomy. The herniated bowel segment was reduced and the mesenteric defect was closed with 2/0 silk sutures (Fig. 2).

The patient’s postoperative course was uneventful after the hernia repair. The patient stayed in the hospital for a few days and was then discharged with adequate oral intake tolerance and normal intestinal transit. Currently, the patient is without complications, leading a normal lifestyle and continuing with controlled weight loss.

## Discussion

Factors associated with internal hernia post Roux-en-Y gastric bypass include weight loss, which thins the mesentery of the small intestine and colon, and the failure to close created defects. The clinical presentation is nonspecific; in our case, it manifested as an immediate complication despite closing the mesenteric defect with continuous non-absorbable suture [3]. The interval between the surgical procedure and the onset of clinical presentation is highly variable; therefore, timely diagnosis and management are crucial due to the risk of bowel strangulation. This highlights the importance of preventive strategies and vigilant postoperative monitoring to optimize patient outcomes [4]. Literature by Stenberg *et al.* reports an incidence of 2.2% for internal hernias with mesenteric defect closure and 7.2% without closure [5].

Traditional techniques for closing Petersen’s space involve the use of sutures or clips. However, these methods have varying success rates and may not entirely prevent herniation. Newer techniques, such as the use of bioabsorbable prosthetic meshes reinforced with fibrin glue, have been developed to promote local adhesions and reduce the risk of hernia recurrence [6, 7]. While traditional methods focus on mechanically closing the defect, newer methods aim to enhance biological healing processes, which could potentially improve long-term outcomes.

The clinical applicability of these techniques can vary depending on the patient population and surgical context. Traditional suture techniques may still be preferred in low-resource settings or when immediate access to advanced materials is limited. In contrast, newer techniques with bioabsorbable meshes may offer better outcomes in specialized centers where such materials are readily available and surgical expertise is high. Continued research and comparative studies are needed to establish standardized protocols and optimize the use of these techniques in clinical practice [8, 9].

Though rare, Petersen’s hernia could lead to a disastrous complication of small bowel strangulation if not intervened early. Despite the closure of Petersen’s mesenteric defect, vigilance is paramount in recognizing and managing Petersen’s space hernia as a potential complication following gastric bypass surgery.

## Conclusions

Even with the closure of Petersen’s mesenteric defect, the risk of hernia remains, necessitating vigilance for early diagnosis and intervention to prevent serious complications like small bowel strangulation. This case underscores the importance of awareness and immediate action in post-bariatric patients with non-specific abdominal symptoms. Advances such as bioabsorbable meshes show promise in reducing recurrence, but further research is needed. Combining prompt diagnosis, effective intervention, and evolving preventive techniques is crucial for improving outcomes in gastric bypass patients.
